# Developmental assets and mental health among black sexual minority male adolescents: A dominance analysis of depressive symptoms and suicidal behaviors

**DOI:** 10.1111/jora.70185

**Published:** 2026-04-19

**Authors:** Donte T. Boyd, Hans Oh, Akilah Patterson, Addie Weaver, Myles Durkee, Camille R. Quinn

**Affiliations:** ^1^ College of Social Work The Ohio State University Columbus Ohio USA; ^2^ School of Social Work University of Southern California Los Angeles California USA; ^3^ Department of Health Behavior and Health Equity, School of Public Health University of Michigan Ann Arbor Michigan USA; ^4^ School of Social Work University of Michigan Ann Arbor Michigan USA; ^5^ Department of Psychology University of Michigan Ann Arbor Michigan USA

**Keywords:** adolescents, depressive symptoms, dominance analysis, suicidal behaviors, young Black sexual minority males

## Abstract

This study examined associations between developmental assets and mental health outcomes among Black sexual minority male (BSMM) adolescents aged 14–17 in the Midwestern United States. Data were collected through an online survey of 383 participants. Dominance analysis was employed to evaluate the relative contributions of multiple developmental assets, including positive identity, social competencies, family support, positive values, and mattering and belonging, as well as depressive symptoms and suicidal behaviors. Results indicated that positive identity demonstrated the strongest relative contribution to both depressive symptoms and suicidal behaviors. Social competencies and family support also contributed meaningfully to variation in suicidal behaviors, whereas mattering and belonging and positive values showed minor but notable contributions to depressive symptoms. Correlation analyses indicated that higher levels of positive identity, social competencies, and family support were associated with lower depressive symptoms and suicidal behaviors. Covariate‐adjusted regression models further indicated that positive identity and mattering and belonging remained significantly associated with suicidal behaviors, whereas positive identity and food insecurity were associated with depressive symptoms. Overall, these findings highlight the importance of both relational and identity‐based developmental assets in promoting mental health among BSMM adolescents. Identifying which assets demonstrate the greatest relative importance provides insights for developing culturally responsive, strengths‐based interventions aimed at reducing suicide risk and improving mental health outcomes among BSMM youth.

## INTRODUCTION

Rates of suicidal thoughts and behaviors (STBs) are disproportionately high among sexual minority adolescents and young adults in the United States (Boyd, Jones, et al., 2024; Boyd, Kirklewski, et al., 2025; Boyd, Quinn, et al., 2025). National surveys consistently reveal an elevated risk of STBs among lesbian, gay, bisexual, transgender, and queer/questioning (LGBTQ+) youth compared to their heterosexual peers. Data from the Youth Risk Behavior Surveillance System indicate that LGBTQ+ students are significantly more likely to report persistent sadness or hopelessness, depressive symptoms, and suicide attempts than heterosexual students (Johns, 2019). Similarly, *The Trevor Project's* 2023 national survey shows that 12% of LGBTQ+ youth reported attempting suicide in the past year, twice that of heterosexual youth, with more than half (56%) reporting depressive symptoms (The Trevor Project, 2024). Together, these data underscore the urgent need for targeted, culturally responsive interventions to address suicide risk and depression among LGBTQ+ populations.

Across the United States, Black youth have experienced a sharp increase in suicide risk. Among Black adolescents aged 10 to 17, suicide rates rose by 144% between 2007 and 2020—a surge that far outpaces the same among other racial and ethnic groups and underscores critical gaps in mental health treatment and access to care (The Pew Charitable Trusts, 2024). Black children aged 5 to 12 are also twice as likely to die by suicide compared to their White peers, and suicide among Black teens is rising at a “shocking” rate (Sheftall, 2023).

Besides the broader mental health crises impacting both LGBTQ+ and Black youth, research focused on young Black sexual minority males (BSMM) has revealed alarming levels of STBs. Among 400 young Black men aged 18 to 29 who have sex with men, 38% reported suicidal ideation, 34% reported having made a suicide plan, and 28% reported a suicide attempt (Boyd, Jones, et al., 2024). A subsequent analysis using the same dataset revealed that family support was associated with lower depressive symptoms and fewer suicide attempts (Boyd, Martinez, et al., 2024). Additional research highlights the role of family communication patterns in shaping suicide planning and attempts among BSMM (Boyd, Feng, et al., 2025) and demonstrates that internal assets—such as positive identity, personal values, and social competencies—are associated with lower levels of STB among BSMM aged 14 to 24 (Boyd, Kirklewski, et al., 2025). Collectively, these findings underscore both the magnitude of suicide risk and the potential impact of developing family‐ and asset‐based suicide prevention approaches for this underserved population.

A growing body of literature focuses on understanding the disproportionate rates of STBs and depressive symptoms experienced by BSMM youth, concentrating on the impact of intersecting oppressions tied to race and sexual orientation (Bostwick et al., 2014; Lucassen et al., 2017). Racial discrimination, internalized stigma, and social isolation contribute to elevated rates of depression and suicidal ideation among Black gay and bisexual men (Earnshaw et al., 2013; Layland et al., 2025). The minority stress model offers an explanatory framework, positing that distal stressors (e.g., discrimination or prejudice) and proximal stressors (e.g., internalized stigma, rejection expectations, or concealment) accumulate to erode mental health (Szymanski & Ikizler, 2013). For BSMM, these stress processes are compounded by racism and gendered expectations, creating unique pathways to depression and suicidality (Szymanski & Ikizler, 2013). Limited access to culturally responsive services and persistent stigma further exacerbate risk, underscoring the need for identity‐affirming interventions addressing the specific needs and experiences of BSMM (Pachankis et al., 2018).

Existing studies addressing BSMM mental health emphasize risk factors, while only a few investigate potential protective factors that could inform targets for prevention and intervention efforts. The limited research examining protective factors for BSMM mental health suggests that developmental assets like family support, positive values, social competencies, belonging, and mattering may buffer the effects of discrimination and foster resilience. This aligns with evidence that developmental assets reduce depressive symptoms and STB in broader youth populations and suggests that examining these strengths among BSMM is critical for identifying protective processes and designing culturally responsive interventions that reduce suicide risk and promote well‐being.

Adolescence and emerging adulthood represent developmental periods marked by rapid changes in identity formation, autonomy development, and shifting reliance on caregivers, schools, and peers (Arnett, 2000; Steinberg & Morris, 2001). For BSMM adolescents, these changes unfold alongside intersecting experiences of racialization, sexual minority stress, and heightened vulnerability to stigma in school and community contexts (Meyer, 2003). Developmental assets theory is particularly relevant during this stage because it conceptualizes protective resources embedded within adolescents' primary ecologies (family, school, and community) and within youths' developing intrapersonal capacities (e.g., positive identity, values, and social competencies) (Benson, 2007; Scales et al., 2000; Syvertsen et al., 2021). Accordingly, this study focuses on assets that are developmentally salient during mid‐adolescence through emerging adulthood and plausible targets for prevention and intervention.

### Developmental assets

Developed in the 1990s by the Search Institute, the Developmental Assets Framework (DAF) is a cornerstone of the positive youth development literature. It shifted attention from deficit‐based models to a strengths‐based approach, emphasizing empirically supported assets that promote adolescent well‐being (Benson, 2007). Developmental assets are broadly categorized into *external* supports in youths' environments (e.g., family support, school engagement, community opportunities, and parental involvement) and *internal* strengths (e.g., social competencies, positive values, positive identity, and future orientation; Benson, 2007; Toomey et al., 2019). Evidence consistently links DAF to better mental health outcomes and reduced suicide risk across adolescence, underscoring its importance for prevention. Yet despite the framework's wide application, asset‐focused research rarely focuses on BSMM, a population disproportionately exposed to intersecting racial and sexual orientation–based oppression and stigma (Bostwick et al., 2014; Earnshaw et al., 2013; Layland et al., 2025; Lucassen et al., 2017). This omission leaves critical gaps in understanding how assets function in the context of structural inequities, community rejection, and the unique cultural realities shaping BSMM lives. Addressing these gaps will clarify whether and how developmental assets buffer against STBs and depression in BSMM, ensuring that the DAF fully reflects the diverse contexts of youth most vulnerable to mental health disparities.

Specific external developmental assets highlighted in the literature include family support and acceptance, which buffer the impact of stigma and discrimination. Caregiver acceptance is linked to higher self‐esteem and lower depression and suicidality, while rejection predicts adverse outcomes (Abreu et al., 2023; Boyd et al., 2021; Boyd, Feng, et al., 2025; Boyd, Sterrett‐Hong, et al., 2025). School belonging, connectedness, and mattering also function as powerful, modifiable protective factors, reducing depression and STB across diverse youth populations (Flett, 2018, 2024; Marraccini & Brier, 2017). Additionally, parental involvement in education reinforces academic engagement and socioemotional well‐being through strengthened family–school partnerships (Allen et al., 2024; Baig et al., 2021; Martinez‐Yarza et al., 2024).

Internal assets like social competencies (e.g., emotion regulation and problem‐solving) and positive values (e.g., integrity) are associated with healthier mental health profiles among youth (Ancín‐Nicolás et al., 2024; Birrell et al., 2025; Busby et al., 2020; Craig et al., 2021; Syvertsen et al., 2021; Toomey et al., 2019). Emerging evidence highlights the importance of internal assets for BSMM (Boyd, Kirklewski, et al., 2025). Specifically, positive identity (comprising self‐esteem, personal power, purpose, and a positive outlook) stands out as a central protective factor for BSMM mental health (Boyd, Kirklewski, et al., 2025). Affirming sexual and racial/ethnic identities has been strongly associated with reduced STBs and depression, and positive identity is among the most empirically supported predictors of adolescent well‐being.

Altogether, studies indicate that both external and internal assets can serve as protective factors against adolescent STBs and depression. For instance, Toomey et al. (2019) and colleagues associated marginalization based on race, sexual orientation, or gender identity with increased risk of STB. Concurrently, they identified internal assets that reduced suicide risk, including planning and decision‐making skills for all youth and social justice values for bisexual adolescents. These assets may buffer against suicidality by supporting healthy choices and fostering a commitment to equity and fairness.

Research on developmental assets among LGBTQ+ youth remains limited, though recent work has begun to acknowledge and address this gap (Fish, 2020; Gonzalez et al., 2020). One study examining suicidal behaviors among Black sexual minority adolescents and young adults found that positive identity, positive values, and social competencies were associated with fewer STB (Boyd, Kirklewski, et al., 2025). These findings posit strengthening internal and external developmental assets as a critical strategy for reducing suicidality and depression across adolescent populations, underscoring the need for continued research on protective factors for LGBTQ+ youth.

Examining developmental assets vis‐à‐vis STBs and depression is especially important for reducing risk among young adults. Such insights can inform culturally responsive interventions that build coping skills, strengthen positive identity, and promote adaptive decision‐making while leveraging assets available through families, schools, and communities. Yet asset‐focused research rarely focuses on BSMM, particularly adolescents in the Midwest, where intersecting racial and sexual orientation‐based stigma, along with regional sociopolitical contexts, may shape both risks and access to supports. Moreover, though often interrelated, developmental assets are typically studied in isolation, limiting clarity about which assets most strongly protect against STBs and depression. Few studies employ dominance analysis to partition explained variance across predictors, making it unclear which assets uniquely and jointly matter the most for BSMM.

### Current study

This study addresses a critical gap in suicide prevention research by focusing on BSMM aged 14 to 17 in the Midwest, a population largely excluded from research on mental health and developmental assets. This developmental period represents a critical window for identity formation, increasing autonomy, and heightened sensitivity to social contexts, making it an important stage for understanding how protective assets may shape mental health trajectories. We examine the relative influence of developmental assets, both external and internal, on reducing STBs and depressive symptoms. Employing dominance analysis, which enables direct comparison of predictors' unique and relative contributions to outcomes of interest, we identify which developmental assets provide the strongest protective effects against STBs and depressive symptoms among young BSMM. Clarifying these pathways, this study advances understanding of how developmental assets collectively operate during a critical stage of adolescence and offers insights for prevention and intervention efforts focused on culturally responsive, strengths‐based approaches. In doing so, this study not only provides novel insights for suicide prevention but also fills a significant gap in the literature by studying a systematically underrepresented population.

## METHODS

### Study procedures, recruitment, and participants

This study employed an online survey to examine the relationship between developmental assets and sexual, physical, and mental health, as well as STBs, among young BSMM aged 14 to 24 residing in three Midwestern cities (Boyd, Kirklewski, et al., 2025; Boyd, Sterrett‐Hong, et al., 2025). Eligibility criteria were consistent across all sampling sites. The survey was created using Qualtrics software, and an anonymous link generated by it was embedded in a recruitment flyer. Paid advertisements were disseminated through social media platforms, including Facebook, X (formerly Twitter) and Instagram. Flyers were also distributed by community‐based organizations and schools, and community health workers shared them with eligible clients. Participants recruited online mostly used personal computers or smartphones, whereas those recruited through community organizations completed the surveys on computers or tablets provided on‐site.

Recruitment occurred between December 1, 2023, and January 31, 2024. Participants under 18 provided assent, and a waiver of parental consent was granted by the Ohio State University Institutional Review Board (#20260144) due to the study's sensitive focus on sexual orientation and mental health and the potential risk of harm if parents were informed of participation. Participants were recruited through a combination of community‐based and online strategies, described above. Approximately 60% of the sample was recruited through schools and community‐based organizations serving adolescents and young adults who self‐identify as LGBTQ+. The remaining participants were recruited through online outreach using social media platforms such as Facebook, Twitter (X), and Instagram. Online recruitment strategies were used to reach adolescents who were not engaged in school‐based programs or community organizations. Eligible participants were individuals assigned male at birth who identified as male, self‐identified as Black or African American, were aged 14 to 24, resided in one of three Midwestern cities, were fluent in English, and reported sexual contact with a male in the past year. Respondents not meeting these criteria were automatically removed from the survey. Eligible participants were granted immediate access to the online survey. To promote data completeness, a response was sought to each item from respondents; however, a “Prefer not to answer” option was available for all questions to maintain participant autonomy and comfort. Participants who completed the 20‐min survey and provided a valid email address received a $35 electronic Visa gift card as compensation.

To maximize data quality and minimize bot activity, the survey employed Qualtrics survey protection features, and the research team verified that respondents' IP addresses were located within the United States. Data integrity was further safeguarded by allowing each respondent to complete the eligibility and survey questions only once. A speeding check excluded participants whose survey completion time was less than one‐third of the median. Additional Qualtrics tools prevented ballot box stuffing, including the placement of a browser cookie upon submission, reCAPTCHA (Completely Automated Public Turing test to tell Computers and Humans Apart) validation, and a survey item that generated a reCAPTCHA score to assess the likelihood of bot activity.

Upon accessing the survey link, respondents completed an informed consent form and a screening tool to determine eligibility. A total of 650 youth and young adults accessed the survey link. Of these, 112 were excluded due to ineligibility (e.g., not meeting age, identity, or sexual behavior criteria), while 538 eligible participants completed the survey. This study's analytical sample comprised 383 participants aged 14 to 17 at the time of survey completion. Older participants (aged 18 and above) were excluded from the study, given the focus on developmental assets and mental health outcomes during adolescence, a period of critical psychosocial and cognitive development. This analysis focuses on survey measures assessing participants' demographics, external and internal developmental assets, and mental health outcomes, including suicidal behaviors and depressive symptoms.

### Participants

Among BSMM adolescents aged 14–17 who completed the survey (M = 15.21, SD = 1.26), most identified as gay (85%), followed by bisexual (12%), queer (2.5%), and asexual (0.5%). Most reported being currently enrolled in school (93%), while 5% were not. Concerning educational attainment, 60.96% of participants were in 12th grade, followed by 11th grade (13.90%), 9th grade or below (8.29%), and 10th grade (6.68%), while 10.16% had obtained a high school diploma or GED. Household income varied across categories, with 36.6% reporting low income (<$40,000), 32.5% medium income ($40,000–$99,999), and 30.9% high income (≥$100,000). Food insecurity was common in the sample, with 51.97% reporting cutting meal size due to financial constraints in the past 12 months.

### Measures

#### Mental health outcomes

Suicidal behavior was assessed using the *Columbia Suicide Severity Rating Scale* (CSSRS), which evaluates suicide attempts, interrupted attempts, and preparatory behaviors. Only the behavioral items were administered, omitting the ideation section. Participants were asked whether they had ever attempted suicide, attempted to do so but were stopped by someone or something (interrupted attempt), or prepared to make an attempt (e.g., wrote a note or obtained means). Each item was dichotomously coded (*Yes*/*No*). A composite suicidal behavior score was created by summing dichotomous items, with higher scores indicating greater engagement in suicidal behaviors. This composite demonstrated strong internal consistency (Cronbach's α = .85), supporting its reliability in assessing suicidal behaviors among adolescents (Conway et al., 2017; Posner et al., 2014).

Depressive symptoms were assessed using the 10‐item version of the Center for Epidemiologic Studies Depression Scale (CES‐D‐10; Andresen et al., 1994), which measures depressive symptoms experienced during the past week. Items include statements such as “I felt depressed” and “I felt hopeful about the future,” rated on a 4‐point scale ranging from 0 (rarely or none of the time) to 3 (all the time). Two items were reverse‐coded before calculating a total score ranging from 0 to 30, with higher scores indicating greater depressive symptom severity. Prior research has examined the psychometric properties of the CES‐D among Black adolescents and suggests that contextual stressors—including exposure to community violence and economic disadvantage—may influence youths' endorsement of certain affective items (Lu et al., 2017). Although these contextual factors were not directly measured in the present study, the CES‐D‐10 demonstrated acceptable internal consistency in this sample (α = .77).

#### Predictors

The developmental asset measures (external and internal) used in this study were adapted from previously validated instruments in the DAF (Benson, 2007; Scales et al., 2000; Syvertsen et al., 2021). Recent work has also applied the framework to examine developmental asset profiles among BSMM adolescents, further supporting the relevance of these constructs in this population (Boyd, Feng, et al., 2025).

##### External developmental assets

Family support was measured using three items adapted from the DAF. Items were assessed on a 5‐point Likert scale ranging from 1 (Strongly disagree) to 5 (Strongly agree). Participants responded to statements such as “My parents help and support me when I need.” Responses were averaged to create a composite score, with higher values indicating greater perceived family support. The scale demonstrated excellent internal consistency in the current sample (Cronbach's α = .95).

Open family communication was assessed with a single item adapted from the DAF. Participants rated the statement “I have lots of good conversations with my parents” on a 5‐point Likert scale ranging from 1 (Strongly disagree) to 5 (Strongly agree), with higher scores reflecting stronger communication.

Communication with parents about sex and drugs was assessed using a single item adapted from the DAF. Participants rated the statement “If you had an important concern about drugs, alcohol, sex, or other serious issues, would you talk to your parent(s) about it?” on a 5‐point Likert scale ranging from 1 (Never) to 5 (All the time), with higher scores indicating greater willingness to communicate with parents about sensitive issues.

Mattering and belonging were assessed using two subscales from the DAF: Caring School Climate (3 items) and Community Values (7 items). Participants responded to items such as “My teachers really care about me” using a 5‐point Likert‐type scale. Because both these subscales capture adolescents' perceptions of mattering and belonging in supportive environments, their items were combined to form a single composite 10‐item indicator, which demonstrated strong internal consistency (α = .85).

Boundaries were measured across three contexts: family (3 items), school (3 items), and neighborhood (1 item). Example items included “In my family, there are clear rules about what I can and cannot do.” Responses were rated on a 5‐point Likert‐type scale ranging from 1 (Strongly disagree) to 5 (Strongly agree). The 7‐item composite demonstrated strong internal consistency (α = .88).

##### Internal developmental assets

Positive identity was assessed using six items on a 5‐point Likert‐type scale, ranging from 1 (Strongly disagree) to 5 (Strongly agree), with higher scores indicating more positive identity. This construct included two subcategories: hope (personal power, sense of purpose, and a positive future outlook) and self‐esteem, e.g., “I feel I do not have much to be proud of” (reverse‐coded), with α = .71.

Positive values were assessed using four subscales: Caring (3 items), Social Justice (2 items), Integrity (2 items), and Responsibility (3 items), for example, “Helping to ensure that all people are treated fairly.” Participants rated their level of agreement with each statement on a 5‐point Likert‐type scale ranging from 1 (Strongly disagree) to 5 (Strongly agree). The combined 10‐item scale demonstrated strong internal consistency (α = .89).

Academic engagement was measured with five items rated on a 5‐point Likert‐type scale ranging from 1 (Strongly disagree) to 5 (Strongly agree), with higher scores indicating higher academic engagement, e.g., “It bothers me when I don't do something well.” In this case, α = .72.

Social competencies included two domains: social–emotional skills (4 items) and planning/decision‐making skills (4 items). Items were rated on a 5‐point Likert‐type scale, an example being “Being good at making and keeping friends.” The scale demonstrated good reliability (α = .88).

#### Covariates

Several sociodemographic variables were included as covariates based on prior research linking structural and economic conditions to adolescent mental health outcomes. Age was measured in years and treated as a continuous variable. Household income was assessed using a categorical item and recoded into three categories: low income (<$40,000), middle income ($40,000–$99,999), and high income (≥$100,000). Low income served as the reference category in regression analyses. Food insecurity was assessed using a standard item asking whether participants had cut the size of meals or skipped meals during the past 12 months due to paucity of money. Responses were coded as a binary variable (0 = no food insecurity, 1 = food insecurity).

#### Analytical plan

Before analysis, data were examined for outliers, normality, and missingness. Missing data were minimal (<3% across variables), and analyses were conducted using listwise deletion. Bivariate correlations were calculated to provide descriptive information regarding associations among developmental assets, mental health outcomes, and sociodemographic variables before conducting dominance and multivariable regression analyses (Table 1).

**TABLE 1:** Pearson correlations among developmental assets and mental health variables (*N* = 383).

Variable	1	2	3	4	5	6	7	8	9	10	11	12	13	14	15	16
1. Suicidal behaviors	–															
2. Depressive symptoms	.38***	–														
3. Parental involvement	−.31***	−.10*	–													
4. Academic engagement	−.18***	.03	.56***	–												
5. Positive identity	−.34***	−.60***	.10*	.23***	–											
6. Positive values	−.30***	−.20***	.66***	.59***	.06	–										
7. Social competencies	−.42***	.01	.59***	.56***	.16**	.76***	–									
8. Family support	−.38***	.03	.77***	.52***	.19***	.67***	.65***	–								
9. Family boundaries	−.13**	−.17***	.60***	.54***	.02	.59***	.57***	.57***	–							
10. Mattering/belonging	−.28***	−.23***	.78***	.51***	−.03	.71***	.63***	.67***	.57***	–						
11. Other adult support	−.27***	−.15**	.72***	.45***	−.02	.66***	.57***	.68***	.50***	.73***	–					
12. Open family communication	−.24***	.10	.72***	.53***	.13**	.62***	.55***	.73***	.62***	.66***	.63***	–				
13. Parent communication (sex/drugs)	.03	−.11*	.50***	.31***	−.04	.45***	.35***	.49***	.43***	.52***	.58***	.54***	–			
14. Age	−.13*	−.16**	.12*	.38***	.30***	.11*	.15**	.12*	.17***	.05	.03	.11*	−.10*	–		
15. Food insecurity	.28***	.07	−.04	.17**	.04	−.09	−.12*	−.09	.01	−.03	−.04	−.01	−.07	.38***	–	
16. Household income	−.03	.04	.05	−.08	−.15**	−.03	−.03	.04	.04	.08	.05	.07	.04	−.45***	−.36***	–

*Note*: Range indicates theoretical scale bounds. All coefficients are Pearson's *r*. *p < .05, **p < .01, ***p < .001. Higher scores indicate greater endorsement of the corresponding construct. Correlations above |.30| are typically interpreted as moderate, and those above |.50| as strong.

To prioritize the relative importance of developmental assets vis‐à‐vis suicidal behaviors and depressive symptoms among BSMM adolescents, dominance analysis was conducted. Dominance analysis is a relative importance method designed for situations in which predictors are conceptually related and statistically correlated. Rather than relying on a single regression model, the approach evaluates each predictor's incremental contribution to explained variance across all possible subset models, allowing predictors to be compared based on their average contribution to model fit (Azen & Traxel, 2009). This method is particularly useful for identifying protective factors that contribute most strongly to variation in outcomes when multiple developmental assets are examined simultaneously.

Fzollowing Azen and Traxel (2009), predictors were compared across all subset regression models to determine their incremental contribution to explained variance. Two complementary dominance metrics were calculated. Conditional dominance analysis (Tables 2 and 3) assessed the relative importance of each asset across models of increasing size, providing insight into the context‐dependent contribution of each predictor. Complete dominance analysis (Tables 4 and 5) evaluated whether one predictor consistently explained more variance than another across all possible model combinations, providing a global ranking of predictor importance.

**TABLE 2:** Conditional dominance analysis for depressive symptoms (*N* = 383).

Variable	Dominance statistic (Δ*R* ^2^)	Standardized dominance	Rank
Parental involvement in schools	0.0044	0.0099	9
Academic engagement	0.0176	0.0397	4
Positive identity	0.3181	0.7195	1
Positive values	0.0284	0.0643	2
Social competencies	0.0163	0.0368	5
Family support	0.0058	0.0132	7
Family boundaries	0.0120	0.0272	6
Mattering and belonging	0.0271	0.0614	3
Other adult support	0.0056	0.0126	8
Open communication about sex and drugs	0.0033	0.0075	11
Open family communication	0.0035	0.0079	10

*Note*: Model fit: Adjusted *R* ^2^ = 0.4421. Dominance statistics represent the average incremental *R* ^2^ contribution of each variable across all possible model combinations. Standardized dominance values (Domin = percent of total *R* ^2^ explained) indicate each variable's relative importance in explaining variance in depressive symptoms. Higher dominance values correspond to greater relative importance.

**TABLE 3:** Conditional dominance analysis for suicidal behaviors (*N* = 383).

Variable	Dominance statistic (Δ*R* ^2^)	Standardized dominance	Rank
Parental involvement in schools	0.0207	0.0585	5
Academic engagement	0.0106	0.0300	10
Positive identity	0.0787	0.2229	1
Positive values	0.0186	0.0526	8
Social competencies	0.0749	0.2122	2
Family support	0.0512	0.1450	3
Family boundaries	0.0140	0.0398	9
Mattering and belonging	0.0189	0.0535	7
Other adult support	0.0194	0.0551	6
Open communication about sex and drugs	0.0099	0.0280	11
Open family communication	0.0362	0.1024	4

*Note*: Model fit: Adjusted *R* ^2^ = 0.4421. Dominance statistics represent the average incremental *R* ^2^ contribution of each variable across all possible model combinations. Standardized dominance values (Domin = percent of total *R* ^2^ explained) indicate each variable's relative importance in explaining variance in depressive symptoms. Higher dominance values correspond to greater relative importance.

**TABLE 4:** Complete dominance analysis for depressive symptoms (*N* = 383).

Variable	*k* = 1	*k* = 2	*k* = 3	*k* = 4	*k* = 5	*k* = 6	*k* = 7	*k* = 8	*k* = 9	*k* = 10	*k* = 11	Average dominance (Δ*R* ^2^)	Rank
Parental involvement in schools	0.0120	0.0081	0.0062	0.0050	0.0041	0.0033	0.0027	0.0023	0.0019	0.0015	0.0012	0.0044	9
Academic engagement	0.0479	0.0332	0.0257	0.0208	0.0169	0.0137	0.0110	0.0088	0.0067	0.0050	0.0033	0.0175	4
Positive identity	0.3545	0.3572	0.3507	0.3412	0.3308	0.3202	0.3095	0.2990	0.2887	0.2786	0.2685	0.3181	1
Positive values	0.0408	0.0337	0.0303	0.0285	0.0273	0.0265	0.0259	0.0255	0.0251	0.0248	0.0245	0.0284	2
Social competencies	0.0003	0.0109	0.0160	0.0186	0.0199	0.0204	0.0203	0.0198	0.0189	0.0177	0.0161	0.0163	5
Family support	0.0015	0.0087	0.0114	0.0114	0.0101	0.0080	0.0059	0.0039	0.0022	0.0009	0.0001	0.0058	7
Family boundaries	0.0308	0.0202	0.0153	0.0127	0.0111	0.0098	0.0087	0.0076	0.0065	0.0054	0.0042	0.0120	6
Mattering and belonging	0.0530	0.0441	0.0379	0.0331	0.0288	0.0250	0.0214	0.0182	0.0151	0.0123	0.0097	0.0271	3
Other adult support	0.0233	0.0126	0.0073	0.0046	0.0031	0.0023	0.0018	0.0016	0.0015	0.0015	0.0015	0.0056	8
Open communication about sex and drugs	0.0105	0.0064	0.0044	0.0033	0.0026	0.0021	0.0018	0.0016	0.0014	0.0013	0.0011	0.0033	11
Open family communication	0.0120	0.0041	0.0016	0.0011	0.0012	0.0016	0.0022	0.0028	0.0034	0.0040	0.0045	0.0035	10

*Note*: Overall model fit (sum of average dominance = total *R* ^2^): *R* ^2^ = 0.4421. Entries *k* = 1 to *k* = 11 are complete dominance values (average incremental Δ*R* ^2^) for each predictor across model sizes. Average dominance is the mean Δ*R* ^2^ across *k* and reflects each variable's overall relative importance under complete dominance. The sum of average dominance values equals the model's total *R* ^2^. Higher values indicate greater relative importance; values <0.10 are small, from 0.10 to 0.30 are moderate, and >0.30 are relatively strong.

**TABLE 5:** Complete dominance analysis for suicidal behaviors (*N* = 383).

Variable	*k* = 1	*k* = 2	*k* = 3	*k* = 4	*k* = 5	*k* = 6	*k* = 7	*k* = 8	*k* = 9	*k* = 10	*k* = 11	Average dominance (Δ*R* ^2^)	Rank
Parental involvement in schools	0.0971	0.0538	0.0300	0.0176	0.0110	0.0072	0.0047	0.0030	0.0018	0.0009	0.0003	0.0207	5
Academic engagement	0.0007	0.0155	0.0182	0.0175	0.0156	0.0134	0.0111	0.0090	0.0070	0.0051	0.0035	0.0106	10
Positive identity	0.1189	0.1074	0.0966	0.0881	0.0808	0.0745	0.0688	0.0638	0.0594	0.0554	0.0520	0.0787	1
Positive values	0.0874	0.0476	0.0262	0.0155	0.0099	0.0065	0.0044	0.0029	0.0018	0.0012	0.0009	0.0186	8
Social competencies	0.1741	0.1252	0.0964	0.0795	0.0685	0.0605	0.0540	0.0484	0.0435	0.0391	0.0351	0.0749	2
Family support	0.1489	0.1008	0.0718	0.0545	0.0433	0.0353	0.0293	0.0247	0.0210	0.0181	0.0158	0.0512	3
Family boundaries	0.0170	0.0102	0.0094	0.0113	0.0133	0.0148	0.0157	0.0160	0.0160	0.0157	0.0151	0.0140	9
Mattering and belonging	0.0813	0.0441	0.0244	0.0149	0.0102	0.0077	0.0063	0.0054	0.0049	0.0045	0.0043	0.0189	7
Other adult support	0.0754	0.0413	0.0236	0.0154	0.0114	0.0094	0.0083	0.0076	0.0073	0.0071	0.0071	0.0194	6
Open communication about sex and drugs	0.0543	0.0245	0.0108	0.0056	0.0037	0.0028	0.0023	0.0018	0.0014	0.0009	0.0005	0.0099	11
Open family communication	0.0008	0.0302	0.0413	0.0451	0.0454	0.0442	0.0423	0.0402	0.0381	0.0361	0.0344	0.0362	4

*Note*: Overall model fit (sum of average dominance = total *R* ^2^): *R* ^2^ = 0.3532. Entries *k* = 1 to *k* = 11 are complete dominance values (average incremental Δ*R* ^2^) for each predictor across model sizes. Average dominance is the mean Δ*R* ^2^ across *k* and reflects each variable's overall relative importance under complete dominance. The sum of average dominance values equals the model's total *R* ^2^. Higher values indicate greater relative importance; values <0.10 are small, from 0.10 to 0.30 are moderate, and >0.30 are relatively strong.

Because dominance analysis is designed primarily as a predictor prioritization method rather than a covariate‐adjusted inference model, additional regression analyses were conducted to examine whether the most influential developmental assets remained associated with outcomes after adjusting for sociodemographic characteristics. Specifically, the four highest‐ranking developmental assets identified through dominance analysis were entered into regression models, adjusting for age, household income, and food insecurity. These models enabled us to assess whether the assets identified as most influential continued to demonstrate significant associations with suicidal behavior and depressive symptoms after considering key demographic factors (Table 6).

**TABLE 6:** Covariate‐adjusted regression models predicting depressive symptoms and suicidal behaviors (*N* = 375).

Predictor	Depressive symptoms (CES‐D 10) B (SE)	*p*	95% CI	Suicidal behaviors B (SE)	*p*	95% CI
Positive identity	−4.09 (0.29)	<.001	[−4.66, −3.51]	−0.88 (0.12)	<.001	[−1.11, −0.64]
Positive values	1.17 (0.40)	.004	[0.38, 1.96]	−0.28 (0.17)	.087	[−0.61, 0.04]
Mattering & belonging	0.52 (0.44)	.237	[−0.34, 1.38]	−0.68 (0.18)	<.001	[−1.04, −0.33]
Academic engagement	0.32 (0.48)	.509	[−0.63, 1.27]	0.33 (0.20)	.092	[−0.05, 0.72]
Age	−0.29 (0.23)	.198	[−0.73, 0.15]	−0.26 (0.09)	.005	[−0.44, −0.08]
Income: Middle ($40 k–$99 k)	−1.44 (0.87)	.099	[−3.16, 0.27]	−0.34 (0.35)	.331	[−1.02, 0.35]
Income: High (≥$100 k)	−1.30 (0.89)	.148	[−3.06, 0.46]	−0.29 (0.36)	.413	[−1.00, 0.41]
Food insecurity	1.40 (0.50)	.006	[0.41, 2.40]	1.33 (0.21)	<.001	[0.92, 1.73]
*R* ^2^	.44			.32		
Adjusted *R* ^2^	.42			.30		

## RESULTS

### Descriptive statistics

Descriptive statistics indicated generally moderate to high levels of developmental assets across domains. Participants reported relatively high levels of family support (*M* = 3.99, *SD* = 0.90), open family communication (*M* = 3.90, *SD* = 1.00), parental involvement in schools (*M* = 3.88, *SD* = 0.90), and positive values (*M* = 3.89, *SD* = 0.90). Mean scores were similarly elevated for social competencies (*M* = 3.83, *SD* = 0.90), mattering and belonging (*M* = 3.77, *SD* = 0.80), other adult support (*M* = 3.75, *SD* = 0.90), and family boundaries (*M* = 3.71, *SD* = 0.80). Lower mean levels were observed for positive identity (*M* = 3.32, *SD* = 0.80) and communication with parents about sex and drugs (*M* = 3.35, *SD* = 1.09). Academic engagement showed the lowest mean score among the assets (*M* = 2.50, *SD* = 1.35). All developmental asset variables were measured on five‐point Likert‐type scales (range = 1–5), with higher scores indicating greater endorsement of each asset.

Participants also reported elevated levels of mental health risk. The mean score on the CES‐D‐10 was 14.35 (*SD* = 5.57), with 80% of participants meeting the threshold for probable depression (CES‐D ≥ 10). Nearly half of the sample (48.9%) met the criteria for moderate or high suicide risk on suicidal behaviors measured by the Columbia Suicide Severity Rating Scale (C‐SSRS), whereas 51.0% were categorized as low or no risk.

### Correlation analysis

Suicidal behaviors were positively associated with depressive symptoms (*r* = .38, *p* < .001) and food insecurity (*r* = .28, *p* < .001), and negatively with several developmental assets, including social competencies (*r* = −.42, *p* < .001), family support (*r* = −.38, *p* < .001), positive identity (*r* = −.34, *p* < .001), and positive values (*r* = −.30, *p* < .001). Depressive symptoms were strongly negatively associated with positive identity (*r* = −.60, *p* < .001) and modestly with mattering and belonging (*r* = −.23, *p* < .001), family boundaries (*r* = −.17, *p* = .001), positive values (*r* = −.20, *p* < .001), other adult support (*r* = −.15, *p* = .003), and age (*r* = −.16, *p* = .002).

Among sociodemographic variables, age was positively associated with academic engagement (*r* = .38, *p* < .001) and positive identity (*r* = .30, *p* < .001). Food insecurity was positively associated with suicidal behaviors (*r* = .28, *p* < .001) and academic engagement (*r* = .17, *p* = .001) and negatively associated with social competencies (*r* = −.12, *p* = .017). Household income was negatively associated with age (*r* = −.45, *p* < .001) and food insecurity (*r* = −.36, *p* < .001).

Consistent with developmental assets theory, several asset constructs were moderately to strongly intercorrelated. For instance, family support was strongly associated with parental involvement in school (*r* = .77, *p* < .001), mattering and belonging (*r* = .67, *p* < .001), and other adult support (*r* = .68, *p* < .001). Social competencies were also strongly associated with positive values (*r* = .76, *p* < .001) and family support (*r* = .65, *p* < .001) (Table 1).

### Conditional dominance analysis

#### Depressive symptoms

Results from the conditional dominance analysis indicated that developmental assets varied substantially in their relative contributions to depressive symptoms (Table 2). Positive identity demonstrated the strongest relative importance (*R* ^2^ = 0.3181; Domin = 71.95%), accounting for the majority of explained variance. Positive values (Δ*R* ^2^ = 0.0284; Domin = 6.43%) and mattering and belonging (*R* ^2^ = 0.0271; Domin = 6.14%) emerged as the next most influential contributors, followed by academic engagement (*R* ^2^ = 0.0176; Domin = 3.97%) and social competencies (*R* ^2^ = 0.0163; Domin = 3.68%). Remaining assets, including family support, family boundaries, and other adult support, demonstrated comparatively smaller contributions (all Δ*R* ^2^s < 0.013).

#### Suicidal behaviors

A parallel pattern emerged for suicidal behaviors, with developmental assets differing in their relative importance (Table 3). Positive identity again demonstrated the strongest contribution (Δ*R* ^2^ = 0.0787; Domin = 22.29%), followed closely by social competencies (Δ*R* ^2^ = 0.0749; Domin = 21.22%) and family support (Δ*R* ^2^ = 0.0512; Domin = 14.50%). Open family communication (Δ*R* ^2^ = 0.0362; Domin = 10.24%) also emerged as a meaningful contributor. Other assets, including parental involvement in schools, other adult support, and mattering and belonging, demonstrated more modest contributions (Δ*R* ^2^ s ranging from 0.0189 to 0.0207), while academic engagement and open communication about sex and drugs contributed minimally.

### Complete dominance analysis

#### Depressive symptoms

Complete dominance analysis indicated clear differences in the relative importance of developmental assets in explaining depressive symptoms (see Table 4). Positive identity emerged as the most influential asset (*R* ^2^ = 0.3181), demonstrating complete dominance over all other predictors across model sizes. Positive values (average Δ*R* ^2^ = 0.0284) and mattering and belonging (*R* ^2^ = 0.0271) represented the next most important contributors, followed by academic engagement (*R* ^2^ = 0.0175) and social competencies (average Δ*R* ^2^ = 0.0163). Remaining assets, including family boundaries (average Δ*R* ^2^ = 0.0120), family support (average Δ*R* ^2^ = 0.0058), and other adult support (*R* ^2^ = 0.0056), demonstrated comparatively smaller contributions. Across predictors, dominance values declined substantially after the top‐ranked asset, indicating that positive identity accounted for the majority of explained variance. The overall model explained 44.21% of the variance in depressive symptoms (*R* ^2^ = 0.4421).

#### Suicidal behaviors

A parallel pattern was observed for suicidal behaviors (see Table 5). Positive identity again emerged as the most influential asset (*R* ^2^ = 0.0787), demonstrating complete dominance across model sizes. Social competencies (*R* ^2^ = 0.0749) and family support (*R* ^2^ = 0.0512) were the next most important contributors, followed by open family communication (*R* ^2^ = 0.0362). Other assets, including parental involvement in schools (*R* ^2^ = 0.0207), other adult support (*R* ^2^ = 0.0194), and mattering and belonging (*R* ^2^ = 0.0189), demonstrated smaller contributions, while academic engagement (*R* ^2^ = 0.0106) and open communication about sex and drugs (*R* ^2^ = 0.0099) contributed minimally. The overall model accounted for 35.32% of the variance in suicidal behaviors (*R* ^2^ = 0.3532).

### Regression analyses

To examine whether the developmental assets identified in the dominance analysis remained associated with mental health outcomes after accounting for sociodemographic characteristics, covariate‐adjusted regression models were estimated (Table 6). Positive identity was strongly associated with lower depressive symptoms (*B* = −4.09, 95% CI [−4.66, −3.51], *p* < .001), food insecurity with higher depressive symptoms (*B* = 1.40, 95% CI [0.41, 2.40], *p* = .006), and positive values with higher depressive symptoms (*B* = 1.17, 95% CI [0.38, 1.96], *p* = .004). For suicidal behaviors, positive identity was associated with fewer suicidal behaviors (*B* = −0.88, 95% CI [−1.11, −0.64], *p* < .001). Mattering and belonging were also negatively associated with suicidal behaviors (*B* = −0.68, 95% CI [−1.04, −0.33], *p* < .001). Age was negatively associated with suicidal behaviors (*B* = −0.26, 95% CI [−0.44, −0.08], *p* = .005), and food insecurity with higher suicidal behaviors (*B* = 1.33, 95% CI [0.92, 1.73], *p* < .001).

## DISCUSSION

### Main findings

This study contributes to a growing body of research examining developmental assets and mental health among BSMM adolescents by identifying which protective assets most strongly relate to suicidal behaviors and depressive symptoms. The findings underscore both the magnitude of mental health risk in this population and the importance of strengths‐based approaches to prevention.

The descriptive findings reveal striking levels of mental health risk among participants. Nearly four out of five adolescents (79.9%) met the criteria for probable depression, and almost half of the sample (48.9%) were classified as having moderate to high risk for suicidal behaviors. These rates substantially exceed national estimates for adolescents and LGBTQ+ youth more broadly (Johns, 2019; The Trevor Project, 2024). The magnitude of these disparities likely reflects the cumulative impact of intersecting stressors tied to racism, sexual minority stigma, and structural inequities that shape the developmental environments of BSMM youth. Together, these findings highlight the urgency of identifying protective processes that may mitigate suicide risk and promote psychological well‐being among BSMM youth navigating multiple forms of marginalization.

The dominance analyses provide new insights into the relative importance of developmental assets in explaining variance in suicidal behaviors and depressive symptoms. For suicidal behaviors, relational and interpersonal assets—particularly social competencies, family support, and positive identity—emerged as the strongest contributors. Conversely, depressive symptoms were most strongly associated with internal psychological assets, with positive identity showing the strongest relationship, followed by mattering and belonging, and positive values. These distinctions suggest that although developmental assets broadly function as protective resources, their relative importance may differ depending on the specific mental health outcome being considered.

Results of regression models adjusted for sociodemographic characteristics further support the assertion that the relative importance of developmental assets may differ across mental health outcomes. That said, positive identity emerged as a developmental asset associated with both fewer suicidal behaviors and lower depressive symptoms after adjusting for sociodemographic characteristics, indicating that it may be a particularly important target for prevention and intervention efforts. These regression models also confirm prior studies suggesting that food insecurity is a risk factor for suicidal behaviors and depressive symptoms.

### Implications for theory

These findings extend the Developmental Assets Framework (DAF) by demonstrating that the relative importance of developmental assets may differ across mental health outcomes among BSMM adolescents. Developmental assets are often conceptualized as broadly protective resources that promote positive youth development (Benson, 2007). However, the present findings suggest that different assets may operate through distinct developmental pathways. Relational assets such as social competencies, family support, and mattering and belonging were most strongly associated with suicidal behaviors in dominance analyses. These assets may reflect adolescents' ability to navigate interpersonal challenges, regulate emotional distress in social contexts, and access support during periods of acute psychological vulnerability. Prior research indicates that supportive relationships and interpersonal coping skills are critical mechanisms through which adolescents manage suicidal crises (Marraccini & Brier, 2017; Toomey et al., 2019).

In contrast, depressive symptoms were most strongly associated with internal assets, particularly positive identity. Adolescence represents a critical developmental period during which identity formation and self‐concept development become central tasks. For sexual minority youth, identity development often occurs in contexts characterized by stigma, discrimination, and social marginalization (Earnshaw et al., 2013; Pachankis et al., 2018). Positive identity, which reflects adolescents' sense of self‐worth, future orientation, and personal value, may therefore function as a core psychological resource protecting against depressive symptoms. Combined, these findings suggest that suicidality and depressive symptoms may reflect partially distinct developmental processes among BSMM adolescents. Integrating the DAF with minority stress theory (Meyer, 2003) may provide a more comprehensive understanding of how protective developmental resources operate within the intersecting structural and social contexts shaping the lives of BSMM youth.

An unexpected finding was that positive values were associated with higher depressive symptoms in covariate‐adjusted models, despite emerging as a protective asset in dominance analyses. One possible explanation is that adolescents who more strongly endorse values related to fairness, caring, and social responsibility may also be more attuned to structural inequities and sociopolitical conditions that conflict with these values. For Black sexual minority male adolescents in particular, heightened awareness of injustice, stigma, and exclusion may contribute to value–context dissonance, which has been linked to increased psychological distress. This interpretation is consistent with research suggesting that moral sensitivity and critical consciousness, while developmentally adaptive, may also confer vulnerability to depressive symptoms in inequitable social environments. Future research should further examine how value endorsement interacts with sociopolitical context to shape mental health among marginalized youth. Taken together, these findings suggest that suicidality and depressive symptoms may reflect partially distinct developmental processes among BSMM adolescents. While positive identity appears to function as a cross‐cutting protective asset, relational assets may be particularly salient for mitigating suicidal behaviors by strengthening interpersonal coping and access to support.

### Limitations

Several limitations should be considered when interpreting these findings. First, participants were recruited through both online advertisements and community‐based channels, which hindered the computation of platform‐specific response rates. Recruitment materials were distributed across multiple digital and community contexts, and analytics were not available for all recruitment channels. Consequently, it was impossible to determine how many individuals viewed recruitment materials on specific online platforms or were recruited via specific community settings, which limited the ability to calculate response rates by recruitment modality. Second, eligibility criteria were based on sexual contact with a male partner rather than sexual identity or attraction alone. This approach was used to capture adolescents engaging in same‐sex relationships regardless of identity labels; however, it may have excluded youth who identify as sexual minorities but have not yet engaged in sexual contact. Because identity exploration is a common developmental process during adolescence, future research should incorporate broader measures of sexual identity, attraction, and behavior to fully capture the diversity of experiences among BSSM. Third, several constructs measuring developmental assets were assessed using single‐item measures, which may limit measurement precision. Although single‐item indicators are sometimes used in adolescent survey research to reduce respondent burden, they may not fully capture the complexity of constructs such as family communication or parental engagement. Future studies must incorporate multi‐item validated measures to strengthen reliability and enable a more nuanced assessment of developmental assets. Finally, the cross‐sectional design limits causal inference. Dominance analysis provides insights into the relative importance of predictors but does not establish causal relationships. Longitudinal research will be necessary to better understand how developmental assets evolve and interact with minority stress processes to shape mental health trajectories among BSMM adolescents. Fourth, although structural and contextual stressors, such as community violence and neighborhood disadvantage, may impact BSMM adolescents' mental health, this study did not measure these constructs. Future research focusing on the relationship between structural and contextual stressors, developmental assets, and mental health outcomes among BSMM adolescents is needed.

### Policy and practice implications

The implications of these findings are particularly salient in the current sociopolitical context in the United States. Schools, families, and community institutions play critical roles in shaping adolescents' developmental environments and fostering developmental assets such as belonging, identity affirmation, and supportive adult relationships. However, recent policy changes in several states have restricted LGBTQ‐inclusive curricula, limited access to gender and sexuality alliances, and weakened protections for LGBTQ students. These changes risk undermining precisely the types of developmental environments that may protect BSMM adolescents from suicide and depression. Strengthening developmental assets among BSMM youth, therefore, requires sustained investment in identity‐affirming school and community environments. Inclusive school climates, enumerated anti‐bullying policies, and LGBTQ student organizations have been shown to reduce victimization and suicidality among sexual minority youth (Johns, 2019). Programs that foster belonging and peer connection may be particularly important, as mattering and belonging were associated with lower suicidal behaviors in the present study (see Chang et al., 2025; Christensen et al., 2025).

Family environments also represent a critical context for intervention (Gay, 2025; Sullivan et al., 2021). Research consistently shows that family acceptance significantly reduces suicide attempts among LGBTQ youth (Ryan et al., 2010). Interventions that support caregivers in developing affirming parenting practices may therefore strengthen protective family processes that support adolescent mental health. Finally, clinical and crisis response systems must prioritize culturally responsive care that affirms both racial and sexual identities (Chang et al., 2025). Identity‐affirming mental health practices improve psychological outcomes among LGBTQ youth (American Psychological Association, 2021; Chang et al., 2025). Expanding access to culturally competent mental health services and strengthening crisis response infrastructure, including services connected to the 988 Suicide and Crisis Lifeline, will be essential for addressing the disproportionate mental health burden experienced by BSMM adolescents.

## CONCLUSION

This study extends the DAF to a critically underrepresented population—BSMM adolescents—and identifies key developmental assets associated with suicidal behaviors and depressive symptoms. The findings highlight the central importance of positive identity as a developmental asset associated with depressive symptoms and suicidal behavior, as well as suggest the specific relevance of relational assets, such as social competencies, family support, and mattering and belonging, as developmental resources that may reduce suicide risk and improve mental health outcomes. Strengthening these developmental assets in supportive family, school, and community contexts represents a promising pathway for promoting resilience and reducing mental health disparities among BSMM youth.

## AUTHOR CONTRIBUTIONS

**Myles Durkee:** Writing – original draft; writing – review and editing; methodology; conceptualization. **Donte T. Boyd:** Conceptualization; methodology; data curation; formal analysis; writing – original draft. **Camille R. Quinn:** Writing – original draft; writing – review and editing; conceptualization. **Addie Weaver:** Writing – original draft; writing – review and editing; methodology; conceptualization. **Akilah Patterson:** Writing – original draft; conceptualization. **Hans Oh:** Writing – review and editing; writing – original draft; conceptualization.

## FUNDING INFORMATION

This research was supported by the National Institute of Mental Health under Grant No. R21MH123456 awarded to Donte T. Boyd.

## CONFLICT OF INTEREST STATEMENT

The authors declare no conflict of interest.

## ETHICS STATEMENT

This study was approved by The Ohio State University Institutional Review Board (IRB# 2023B0242).

## PATIENT CONSENT STATEMENT

Patient consent statement is not applicable.

## Data Availability

The data that support the findings of this study are available from the corresponding author upon reasonable request.
